# Iron isotopes trace primordial magma ocean cumulates melting in Earth’s upper mantle

**DOI:** 10.1126/sciadv.abc7394

**Published:** 2021-03-12

**Authors:** Helen M. Williams, Simon Matthews, Hanika Rizo, Oliver Shorttle

**Affiliations:** 1 Department of Earth Sciences, The University of Cambridge, Cambridge, UK.; 2 Department of Earth Sciences, Carleton University, Ottawa, ON, Canada.; 3 Institute of Astronomy, The University of Cambridge, Cambridge, UK.

## Abstract

The differentiation of Earth ~4.5 billion years (Ga) ago is believed to have culminated in magma ocean crystallization, crystal-liquid separation, and the formation of mineralogically distinct mantle reservoirs. However, the magma ocean model remains difficult to validate because of the scarcity of geochemical tracers of lower mantle mineralogy. The Fe isotope compositions (δ^57^Fe) of ancient mafic rocks can be used to reconstruct the mineralogy of their mantle source regions. We present Fe isotope data for 3.7-Ga metabasalts from the Isua Supracrustal Belt (Greenland). The δ^57^Fe signatures of these samples extend to values elevated relative to modern equivalents and define strong correlations with fluid-immobile trace elements and tungsten isotope anomalies (μ^182^W). Phase equilibria models demonstrate that these features can be explained by melting of a magma ocean cumulate component in the upper mantle. Similar processes may operate today, as evidenced by the δ^57^Fe and μ^182^W heterogeneity of modern oceanic basalts.

## Teaser

Iron isotope data for ancient basalts can be explained by melting of magma ocean cumulate component(s) in the upper mantle.

## INTRODUCTION

Earth’s formation is thought to have been dominated by planetary-scale collision and melting events, culminating with the formation of a magma ocean following the moon-forming impact (*1*). Models of magma ocean cooling and crystallization have been widely invoked to explain initial conditions and the internal structure of Earth’s mantle but remain difficult to test with geological observations. A key archive of this stage of Earth history is the crystal cumulate piles and domains of residual trapped melt that magma ocean solidification would produce (*2*–*4*). These may act as important reservoirs in the mantle for heat-producing elements (e.g., U, Th, and K) and, as a consequence of crystal chemistry, may have notable oxidizing capacity (*5*). Some of these mantle “reservoirs” or domains might have remained isolated from the convective processes that have homogenized much of Earth’s mantle. The preservation of “primordial” geochemical signatures (e.g., high ^3^He/^4^He) in rare basalts associated with mantle plumes has resulted in the paradigm of chemically isolated and undegassed mantle reservoirs, often presumed to reside in the lower mantle (*6*). Geophysical observations of large low shear-velocity provinces and ultralow shear-velocity provinces provide evidence, at the base of the lower mantle, for the existence of thermochemical piles that are different from ambient mantle (*7*), potentially residual from early magma ocean crystallization. However, the composition, mineralogy, and hence origin of this material remain undetermined.

Evidence for the preservation of ancient mantle chemical heterogeneity has been reinforced by recent geochemical measurements (e.g., of the noble gasses and of resolvable variations in the daughter isotopes of short-lived ^146^Sm-^142^Nd and ^182^Hf-^182^W isotope systems, reported as μ^142^Nd and μ^182^W, respectively) of ancient [> 2.5 billion years (Ga) old] and modern mantle melts [e.g., (*8*–*12*)] that could be placed in the framework of derivation from lower mantle domains formed from the cooling and crystallization of an early magma ocean. However, these observations cannot in themselves constrain the mineralogy and composition of mantle source regions, such that it remains difficult to constrain the source mineralogy of primitive mantle melts with μ^142^Nd and μ^182^W anomalies and hence link mantle mineralogical heterogeneity to the differentiation history of Earth.

Detecting the mineralogical signature of lower mantle material in the source regions of primitive magmas using traditional geochemical and petrological tools is challenging. Phase stoichiometry determines that lower mantle phases such as bridgmanite, calcium perovskite, and ferropericlase transform to orthopyroxene, clinopyroxene, and majorite garnet and olivine when transported to the upper mantle. These assemblages would display similar melting behavior to that of ambient upper mantle peridotite such that, even if lower mantle material could be transported to the upper mantle and undergo melting, the products would be extremely difficult to detect using conventional tracers.

Novel stable isotope systems based on major elements such as iron are powerful tools that can be applied to this problem because they record the major phase assemblage properties of mantle materials. Iron (Fe) is stoichiometrically incorporated into upper and lower mantle phases, the latter including bridgmanite and ferropericlase as well as metallic iron (Fe^0^). Advances in mass spectrometry and our understanding of Fe isotope partitioning at high pressures and temperatures, including experimental studies, now make it possible to apply this system to the deep mantle (*13*–*22*). Theory and experimental studies and both predict that isotopically “heavy” Fe (high δ^57^Fe, per mil deviation in ^57^Fe/^54^Fe relative to a standard) may be associated with bridgmanite and low-spin ferropericlase (*14*–*15*, *19*), whereas isotopically low or “light” Fe (low δ^57^Fe) preferentially partitions into metallic iron relative to these crystalline silicate phases (*14*). In contrast, minimal Fe isotope fractionation is expected to take place between silicate melt and molten iron metal (*16*, *19*–*20*).

In this study, we apply the Fe stable isotope system to 3.7-Ga metabasalts from the Isua Supracrustal Belt (ISB), which display radiogenic isotope signatures that have been used to argue for a deep mantle origin (*9*). We use our data in conjunction with phase equilibria modeling to test the hypothesis that the mantle source of the ISB metabasalts contains a component derived from the melting of a lower mantle cumulate component, which was then transported and remelted in the upper mantle. We then consider the application of our results to mantle source heterogeneity as expressed in the geochemistry of modern ocean island basalts (OIB) that display μ^182^W and noble gas isotope evidence for a primordial, undegassed mantle source regions.

## RESULTS

### Sample details

We analyzed a series of well-preserved and well-characterized tholeiitic basalts metamorphosed to amphibolite facies, from the 3.72-Ga Northern Terrane of the ISB for their bulk Fe isotope compositions (see Materials and Methods for more details). These samples define isochrons in ^147^Sm/^144^Nd-^143^Nd/^144^Nd and ^176^Lu/^177^Hf-^176^Hf/^177^Hf space that yield geologically meaningful ages within error of each other [3716 ± 84 million years (Ma) and 3674 ± 70 Ma, respectively; (*9*)], demonstrating that both long-lived Sm-Nd and Lu-Hf systems behaved as closed systems after emplacement. A key feature of the ISB samples studied here, revealed by the ^147^Sm/^144^Nd-^143^Nd/^144^Nd and ^176^Lu/^177^Hf-^176^Hf/^177^Hf isochrons, is their decoupled initial ^176^Hf/^177^Hf (subchondritic) and ^143^Nd/^144^Nd (superchondritic) ratios (*9*). It is widely known that bridgmanite is capable of decoupling the Hf and Nd isotope systems (*23*–*24*), as it is the only silicate phase found so far in which Hf is more compatible than Lu, whereas Sm is more compatible than Nd in all mantle silicates (*25*–*27*). The decoupled initial ^176^Hf/^177^Hf-^143^Nd/^144^Nd ratios of the ISB samples, in conjunction with the presence of positive ^142^Nd anomalies relative to a terrestrial standard, have thus been explained by derivation from a bridgmanite–ferropericlase–calcium perovskite cumulate that formed in the first ~500 Ma of Earth history (*9*–*12*, *28*). The ISB samples also display positive ^182^W anomalies, which can be explained by Hf/W crystal-liquid fractionation within the first ~45 Ma of solar system history, while ^182^Hf was still extant (*10*).

### Iron isotopes

**Fig. 1:**
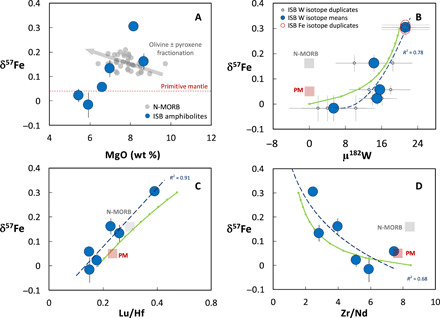
Covariation of measured δ^57^Fe values with elemental and isotopic tracers. (**A**) δ^57^Fe vs. MgO, (**B**) δ^57^Fe vs. μ^182^W, (**C**) δ^57^Fe vs. Lu/Hf, and (**D**) δ^57^Fe vs. Zr/Nd. Large blue circles show the Isua samples analyzed for Fe-isotopes, with 2 SD error bars. ISB, Isua Supracrustal Belt samples studied [Northern Terrane 3.72-Ga suite; (*9*, *10*); PM, primitive mantle; MORB, average mid-ocean ridge basalts; see (*21*) and Materials and Methods for sources of iron isotope and trace element data, respectively]. The dashed blue lines are regression curves fitted to the data; *R*^2^ values are shown for reference. The green model curves in (B) to (D) show mixing calculations between a 50% upper mantle melt of the cumulate-derived lithology and a 30% melt of depleted upper mantle peridotite (model details given in the main text and Materials and Methods). Ticks show mixing proportions in 10% intervals.

The Fe isotope compositions of the ISB samples are given in Table 1 and range from values of −0.02 ± 0.05 per mil (‰), which overlap with current estimates for the δ^57^Fe of the primitive mantle (*18*, *21*, *29*), to values of 0.30 ± 0.01‰, which are heavier than the majority of OIB or mid-ocean ridge basalts (MORB) sampling the modern mantle (which both average δ^57^Fe ~0.15‰, with respective ranges of 0.4 and 0.2‰; *21*, *29*). This range in δ^57^Fe is considerable and cannot be explained by the posteruptive mobility of Fe (e.g., during later metamorphism and alteration; see Materials and Methods for further discussion) and is far greater than can be produced by any reasonable degree of partial melting of a source of homogeneous Fe isotope composition (*21*). Although good correlations between δ^57^Fe and major element contents such as MgO exist (Fig. 1A), they cannot be explained by igneous processes such as olivine and pyroxene fractionation, as previous studies have shown that these produce trends where the most evolved samples display the heaviest δ^57^Fe (*13*, *17*, *18*, *21*, *29*–*32*), opposite to what is observed (Fig. 1). While it is clear that none of the measured samples are sufficiently primitive to have been in equilibrium with the mantle, crustal assimilation and magmatic differentiation can also be ruled out as explanations for the unusual heavy δ^57^Fe signatures, as these are observed in the more primitive samples (Fig. 1A). In Fig. 1B, we observe a strong correlation between δ^57^Fe and μ^182^W (*R*^2^ = 0.78 using a second-order polynomial fit; the *R*^2^ for a simple linear fit is 0.64), with the samples having the largest positive μ^182^W values also displaying heavy δ^57^Fe. The same correlation is also present using calculated “primitive” Fe isotope compositions [where sample Mg# and Fe isotope composition are corrected for olivine-only fractionation to be in equilibrium with Fo_90_ olivine; see Materials and Methods; (*31*)]. Although the distribution—and potential homogenization—of μ^182^W values in ancient rocks has been linked to the mobilization of W by oxidizing fluids (*33*), no correlations between δ^57^Fe and W concentration or W/Th ratio (a proxy for W mobility) are observed, suggesting that fluid-induced modification of δ^57^Fe and μ^182^W was limited in extent. Furthermore, strong correlations exist between δ^57^Fe and fluid-immobile trace element ratios such as Lu/Hf (*R*^2^ = 0.91, linear fit; Fig. 1C) and Zr/Nd (*R*^2^ = 0.68, logarithmic fit; *R*^2^ = 0.56 linear fit; Fig. 1D), where samples with the most pronounced depletions in the high-field strength elements (HFSE) Hf and Zr (high Lu/Hf and low Zr/Nd, respectively) display the heaviest δ^57^Fe. The correlations between δ^57^Fe and trace element ratios involving HFSEs, which are generally fluid immobile and hence resilient to metasomatic and metamorphic overprinting, further demonstrate the robust nature of δ^57^Fe as a tracer in ancient rocks.

**Table 1:** Iron isotope and published major, trace element, μ^142^Nd, and μ^182^W data for 3.7 North Terrane ISB samples. LOI, loss on ignition.

**Sample name**	**00-007**	**00-008**	**00-010**	**00-012**	**00-042**	**00-044**
SiO_2_ (wt %)	55.30	52.86	51.04	49.92	50.44	51.58
Al_2_O_3_	14.07	14.71	15.14	14.81	15.80	13.18
Fe_2_O_3_^total^	11.90	11.95	12.35	12.88	10.03	13.88
MgO	5.45	5.92	6.60	6.98	8.67	8.16
CaO	8.88	9.12	10.48	10.04	11.64	9.22
Na_2_O	2.89	3.81	2.60	3.85	2.59	2.38
K_2_O	0.28	0.45	0.55	0.32	0.11	0.33
TiO_2_	0.90	0.83	0.88	0.94	0.49	1.03
MnO	0.18	0.20	0.18	0.17	0.13	0.19
P_2_O_5_	0.13	0.13	0.15	0.05	0.06	0.04
LOI	1.18	0.52	0.47	0.20	0.63	0.29
Al/Fe*	0.89	0.93	0.93	0.87	1.19	0.72
Mn/Fe*	0.02	0.02	0.02	0.01	0.01	0.02
Nd	10.59	9.80	14.24	4.33	3.38	11.89
Zr	54.20	57.80	106.30	12.20	13.50	29.20
Hf	2.35	2.09	2.91	0.82	0.82	1.56
Lu	0.42	0.31	0.44	0.21	0.19	0.61
Lu/Hf	0.18	0.15	0.15	0.26	0.23	0.39
Zr/Nd	5.12	5.90	7.46	2.82	3.99	2.46
μ^142^Nd	8.3	9.4	4.5	16.3	2.6	8.2
±	2	1.4	1.5	4.1	7.7	4.8
μ^182^W	15.0	5.4	15.6	–	14.3	21.3
±	3.1	7.2	6.8	–	8.5	5.3
**δ^57^Fe**	**0.02**	**−0.02**	**0.06**	**0.13**	**0.16**	**0.30**
±2 SD	0.03	0.05	0.02	0.03	0.03	0.01
δ^56^Fe	**0.02**	**−0.01**	**0.04**	**0.10**	**0.11**	**0.21**
±2 SD	0.02	0.01	0.02	0.02	0.01	0.03
*n*	*5*	*6*	*3*	*6*	*4*	*8*
**δ^57^Fe**		*(replicate dissolution of sample 00-044)*				**0.31**
±2 SD						0.01
δ^56^Fe						**0.23**
±2 SD						0.02
*n*						*2*
**δ^57^Fe**		*(replicate dissolution of sample 00-044)*				**0.30**
±2 SD						0.02
δ^56^Fe						**0.19**
±2 SD						0.03
*n*						*6*
δ^57^Fe_Mg#74_^†^	−0.07	−0.10	−0.01	0.06	0.13	0.24
±2 SD	0.03	0.05	0.02	0.03	0.03	0.01

*Calculated using standard atomic masses and assuming all Fe present as Fe^2+^.

†Calculated by olivine addition [e.g., (*31*, *62*)].

Other explanations are therefore required to explain the substantial range in δ^57^Fe observed, and, in particular, the heavy δ^57^Fe of the most primitive samples. Given that igneous processes, crustal assimilation, and postmagmatic alteration can be ruled out, we can now consider explanations that relate to the Fe isotope composition or lithology of the mantle source region from which these magmas were derived. It has also been proposed that pyroxenite lithologies melting within the mantle can create melts with heavy δ^57^Fe signatures as a consequence of mineral-specific isotope partitioning effects [e.g., (*21*)]. However, mantle pyroxenite components are readily identified through positive correlations between δ^57^Fe and radiogenic isotope signatures indicative of crustal origins (*31*, *32*), and these are not observed in the ISB samples. Direct entrainment of heavy-δ^57^Fe crustal material into the mantle source region(s) sampled by the ISB melts is also an unlikely explanation due to the high concentration of Fe in the mantle relative to oceanic crust and sediments.

Experimental and theoretical studies suggest that the lower mantle phases bridgmanite and low-spin ferropericlase may preferentially incorporate isotopically heavy Fe relative to high-spin ferropericlase and iron metal (*14*, *15*, *19*). Although the exact magnitude of this isotope partitioning at the relevant conditions is not well known, the overall direction of fractionation suggests that the lower mantle will acquire a heavy δ^57^Fe signature as a consequence of the bridgmanite-driven disproportionation of FeO into Fe^3+^ (which partitions into bridgmanite) and Fe^0^ metal, which is removed to the core. A lower mantle (bridgmanite and calcium perovskite–bearing cumulate) origin for the source region of the ISB samples has also been proposed on the basis of their decoupled initial ^143^Nd/^144^Nd and ^176^Hf/^177^Hf signatures and positive μ^182^W and μ^142^Nd values (*9*, *10*). In this context, the most straightforward interpretation of the ISB heavy δ^57^Fe values and positive δ^57^Fe-μ^182^W correlation in Fig. 1A is that the latter represents the mixing of melts derived from two endmember domains, one with δ^57^Fe and μ^182^W signatures close to those inferred for the primitive upper mantle (δ^57^Fe = 0.04‰, μ^182^W = 0 by definition) and the other with positive μ^182^W and elevated δ^57^Fe, originally derived from the lower mantle, but now melting in the upper mantle.

Critically, any such model must also be able to resolve the decoupled initial ^143^Nd/^144^Nd and ^176^Hf/^177^Hf ratios of the ISB samples with their μ^142^Nd values, major and trace element compositions. Melting an upper mantle source region that formed from a bridgmanite cumulate residual from magma ocean crystallization should yield melts with demonstrable enrichment in HFSE relative to elements of similar compatibility, due to the affinity of HFSE for bridgmanite and their relative incompatibility in major upper mantle silicate phases (orthopyroxene, clinopyroxene, garnet, and olivine). However, the ISB samples display depletions in HFSE relative to elements of similar incompatibility rather that the enrichment that would be expected if they were derived from the direct melting of a bridgmanite cumulate in the upper mantle, where it would undergo phase change to an orthopyroxene-dominated lithology. This is evident in Fig. 1 (C and D), where δ^57^Fe displays a strong positive correlation with Lu/Hf and a negative correlation with Zr/Nd.

Is it possible to reconcile the radiogenic isotope and trace element signatures of the ISB with scenarios that do not involve the involvement of lower mantle material? Explanations for HFSE depletions and decoupled initial ^143^Nd/^144^Nd and ^176^Hf/^177^Hf ratios observed in other ISB suites include a subduction origin (*34*), but this does not account for the strong correlations present between δ^57^Fe, positive μ^182^W, and fluid-immobile trace element ratios. Moreover, a subduction scenario is inconsistent with the heavy δ^57^Fe of the ISB, as arc lavas display almost uniformly light δ^57^Fe, which is generally considered to reflect their derivation from a comparatively depleted and light-δ^57^Fe mantle wedge (*30*). It has also been suggested that melts derived from pyroxenite mantle lithologies formed by crustal recycling should display heavy δ^57^Fe, due to mineral-specific Fe isotope partitioning effects and the elevated δ^57^Fe of likely pyroxenite protoliths [oceanic crust; MORB; (*21*, *29*)]. However, even if crustal recycling was operating well before 3.7 Ga, a recycled pyroxenite source lithology should be preferentially enriched in incompatible elements, inconsistent with the positive μ^182^W and μ^142^Nd anomalies displayed by the ISB, which require time-integrated depletion in incompatible elements (W and Nd relative to Hf and Sm). Moreover, a crustal recycling scenario cannot explain the decoupled initial ^143^Nd/^144^Nd and ^176^Hf/^177^Hf ratios of the ISB.

Studies of natural samples and experimental work [e.g., (*13*, *17*, *21*, *22*)] have demonstrated that garnet preferentially incorporates light Fe isotopes relative to pyroxene and olivine. Melting in the garnet stability field has therefore been proposed as a mechanism for generating primitive melts with heavy δ^57^Fe (*13*, *31*). However, new mantle melting models incorporating phase equilibria and Fe isotope force-constant data for different mantle minerals predict that melts generated in the garnet stability field will have δ^57^Fe values almost indistinguishable from those of spinel-facies melts (*32*). Furthermore, melting in the presence of residual garnet should result in a negative correlation between δ^57^Fe and Lu/Hf, due to the greater compatibility of Lu in garnet relative to Hf, which is opposite to what is observed (Fig. 1C).

A deep-seated (lower mantle) origin appears to be the most straightforward explanation for the heavy δ^57^Fe-positive-μ^182^W endmember of the ISB samples, as well as the decoupled initial Nd-Hf isotope signatures of the entire suite. In this scenario of mixing between a heavy δ^57^Fe lower mantle–derived component, now melting in the upper mantle, and ambient upper mantle (δ^57^Fe ~ 0.04), the ^147^Sm/^144^Nd-^143^Nd/^144^Nd and ^176^Lu/^177^Hf-^176^Hf/^177^Hf isochrons date the concurrent mixing and fractional crystallization of these melts at 3.72 Ga and the initial ^143^Nd/^144^Nd and ^176^Hf/^177^Hf values obtained from isochron regression record the overall Nd and Hf isotope compositions of the melt mixture at the time of crystallization. Any proposed model used to explore this scenario must reconcile all the stable, radiogenic, and extinct isotopic signatures of these samples in addition to their trace element geochemistry. We expand on this below, using a phase equilibria approach (*35*–*37*) coupled with trace element and stable isotope modeling.

### Modeling constraints on the nature of the ISB mantle source region

Quantitative melting models are essential to test the hypothesis that lower mantle bridgmanite-dominated cumulates are involved in the generation of the ISB basalts via melting in the upper mantle. Furthermore, incorporating trace element ratios and Fe isotopes into these models provides us with a view of the processes leading to the ascent of deep-seated mantle source regions into the upper mantle, in addition to that of the long-term cumulate storage and magma ocean crystallization processes proffered by ingrown μ^182^W, μ^142^Nd, ^143^Nd/^144^Nd, and ^176^Hf/^177^Hf.

**Fig. 2:**
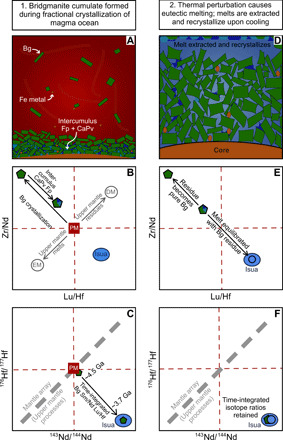
Schematic demonstration of how the mantle component sampled by Isua basalts may have been formed. (**A** to **C**) Show the formation of the initial bridgmanite (Bg) cumulate with a small amount of interstitial calcium perovskite (CaPv) and ferropericlase (Fp). (**D** to **F**) Demonstrate the chemical effects after a eutectic melt is extracted from the cumulate before recrystallizing as a new assemblage in the lower mantle. (B), (C), (E), and (F) show the geochemistry of the Isua samples relative to primitive mantle (PM) as a blue ellipse. EM, enriched mantle; DM, depleted mantle.

We have constructed a series of models that recreate the main major and trace element features of the Isua samples in conjunction with their decoupled Nd-Hf radiogenic isotope and heavy δ^57^Fe signatures. The key features of the data that we seek to recreate in our models are (i) the elemental and radiogenic isotopic signatures of the heavy δ^57^Fe and positive μ^182^W endmember and (ii) the apparent mixing arrays between this endmember and the ambient upper mantle endmember (Fig. 1, B to D). In our models, we calculate the trace element concentrations of magmas composed of a mixture of melt from a lower mantle cumulate–derived lithology (the heavy-δ^57^Fe, positive-μ^182^W endmember) and melt from a depleted upper mantle component; note that the models do not incorporate the effects of fractional partial melting in the upper mantle as this would generate minimal change in both Fe isotope composition (discussed further in Materials and Methods) and incompatible element ratios. The results are shown as curves superimposed on the data in Fig. 1 (B to D). A schematic of the scenario that we envisage forming the heavy-δ^57^Fe, positive-μ^182^W endmember is shown in Fig. 2. Our preferred scenario is discussed in detail below, and our modeling approach is provided in Materials and Methods.

A multistage process is required to account for the trace element (HFSE depleted) and radiogenic isotope (low time-integrated Lu/Hf) signatures of ISB samples. The trace element and isotopic signatures of this endmember can be reconciled with our current understanding of magma ocean crystallization processes via a three-stage process (shown schematically in Fig. 2; model details in Materials and Methods); the feasibility of such a scenario is explored below. In the first stage, a bridgmanite residue is formed during a single stage of magma ocean fractional crystallization. We used a 95% bridgmanite and a 5% calcium perovskite assemblage as required by ^143^Nd/^144^Nd and ^176^Hf/^177^Hf constraints (*9*). The magma ocean from which these phases crystallized was assumed to have the composition of the bulk silicate Earth (BSE) after core formation, and the resulting bridgmanite-dominated cumulate is relatively enriched in HFSE (i.e., high Zr/Nd and low Lu/Hf relative to the BSE; see Fig. 2) because of their compatibility in this phase. The partition coefficients determined by Corgne *et al*. (*25*) for bridgmanite and calcium perovskite were used throughout the calculations for consistency (Table 2; Materials and Methods); we note that they are in excellent agreement with those determined by Walter *et al*. (*27*) and Liebske *et al*. (*26*). Although we might expect some component of ferropericlase to contribute to the cumulate, this has little influence on trace element ratios [or indeed, radiogenic isotopes, as noted in (*9*)] and was not included in the calculations.

**Table 2:** Partition coefficients used for modeling trace element fractionations between minerals and magmas in the upper and lower mantle. The initial concentration of elements in the magma ocean is given by C_0_ (*27*). ppmw, parts per million by weight.

	**Lu**	**Hf**	**Zr**	**Nd**
C_0_ (ppmw)	0.064	0.19	8.26	0.81
Bridgmanite*	0.73	1.3	1.38	0.016
Ferropericlase^†^	0.11	0.076	0.15	0.036
Ca perovskite*	11	1.3	1.6	22
Upper mantle^‡^	0.12	0.035	0.033	0.031

*Corgne *et al*. (*25*).

†Walter *et al*. (*27*).

‡Workman and Hart (*68*).

In our proposed scenario, the second stage takes place shortly before ~3.7 Ga (the emplacement age of the ISB lavas) and involves the eutectic-like melting of the prior-formed cumulate leaving behind a bridgmanite (± ferropericlase) residue (Fig. 2). Crystallization of the eutectic cumulate-derived melt would result in it becoming frozen in the lower mantle near its presumed source. Subsequent mantle upwelling then entrains this frozen melt, transporting it to the upper mantle. Our models show that this melt component would have a lower solidus than nominally anhydrous mantle peridotite when transported to upper mantle conditions. In contrast, the nearly pure bridgmanite (± ferropericlase) cumulate residue from which this melt was originally extracted would be highly refractory, as bridgmanite ± ferropericlase undergoes phase transformation to an orthopyroxene ± olivine lithology in upper mantle conditions (see Materials and Methods for details).

Critically, the second-stage eutectic melt component discussed above crystallizes in a closed system to produce a new lower mantle component. It inherits ingrown and decoupled ^143^Nd/^144^Nd and ^176^Hf/^177^Hf ratios as a consequence of bridgmanite crystallization in stage 1 but develops fractionated Sm/Nd, Zr/Nd, and Lu/Hf ratios to the original stage 1 cumulate, as consequence of mineral-melt partitioning and the compatibility of the HFSE in bridgmanite (Fig. 2). In stage 3, this crystallized melt component is then extracted and transported to the upper mantle during subsequent mantle upwelling events, where it ultimately undergoes melting alongside ambient upper mantle peridotite in the source region sampled by the Isua metabasalts.

**Fig. 3:**
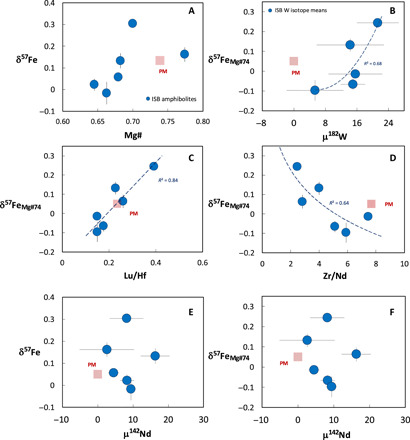
Covariation of measured δ^57^Fe values with Mg# and μ^142^Nd, and δ^57^Fe values corrected to Mg# of 74 (Table 1; δ^57^Fe_Mg#74_) with μ^182^W, Lu/Hf, Zr/Nd, and μ^142^Nd. Large blue circles show the Isua samples analyzed for Fe isotopes, with 2 SD error bars. ISB, Isua Supracrustal Belt samples studied (Northern Terrane 3.72-Ga suite; *11*); PM, primitive mantle.

Our models demonstrate that the presence of a lower mantle component is required to explain the HFSE ratios of the heavy-δ^57^Fe, positive-μ^182^W samples, in conjunction with the decoupled initial ^143^Nd/^144^Nd and ^176^Hf/^177^Hf ratios of the entire ISB suite. As discussed further below, our models provide a mechanism that can explain the heavy δ^57^Fe and positive μ^182^W of this lower mantle material. Furthermore, our models demonstrate that the concurrent melting and binary mixing of this lower mantle component with melts from the spinel stability field of the upper mantle (Fig. 1C) must have taken place to produce the observed covariations between δ^57^Fe and Zr/Nd and Lu/Hf (Fig. 1, C and D). We note that the ISB samples all display similar μ^142^Nd values, within uncertainty, and that no correlation exists between δ^57^Fe and μ^142^Nd (Fig. 3; Materials and Methods). In the context of our model, the absence of any correlation between δ^57^Fe and μ^142^Nd provides evidence for Sm/Nd fractionation and melt extraction before mixing and during the generation of both the heavy-δ^57^Fe bridgmanite cumulate and the ambient upper mantle (now presumed to be depleted mantle) components. While it is likely that the magnitude and timing of Sm/Nd fractionation during the formation of these components were different, the limited variation in sample μ^142^Nd, relative to analytical uncertainties, does not allow this to be further constrained.

## DISCUSSION

Our new Fe isotope data and models demonstrate that the ISB basalts are the surface expression of the mixing and differentiation of two distinct mantle components at 3.7 Ga, one with δ^57^Fe-μ^182^W signatures and a μ^142^Nd value suggestive of depleted upper mantle, the other with heavy δ^57^Fe and positive μ^182^W. We suggest that the former component represents upper mantle that was depleted in the first ~500 Ma of Earth history, now melting in the garnet stability field (generating melts with low Lu/Hf; Fig. 1C) and that the latter component was formed from a mixture of depleted upper mantle and a component derived from the lower mantle. We envisage that the lower mantle component is a solidified eutectic melt formed from the melting of bridgmanite and calcium perovskite cumulate formed during early magma ocean crystallization, and this was subsequently extracted to the upper mantle, potentially in a separate melting event, leaving behind a highly refractory, nearly pure bridgmanite residue. The ISB samples therefore provide a tantalizing glimpse of the residues from magma ocean crystallization that formed within the first ~45 Ma of Earth history and the means by which they may be subsequently sampled by upper mantle melting processes.

The heavy δ^57^Fe and positive μ^182^W signatures of the inferred lower mantle component of the ISB place constraints on the timing and nature of the internal mantle differentiation processes in operation by 3.7 Ga. Tungsten anomalies (in modern and ancient rocks) require the fractionation of Hf from W while ^182^Hf was still extant. The causes of the μ^182^W enrichments seen in ISB samples remain controversial. One possibility is that the ISB mantle source remained relatively isolated from late accretionary additions of materials with chondritic μ^182^W (*38*) and preserves a tungsten isotope composition inherited from Hf/W fractionation during core formation. Late (after core formation) accretion of metal-rich meteoritic material has also been proposed as a mechanism to explain excesses in mantle μ^182^W, as W is extracted into the newly added metal and locally removed from the mantle, effectively raising Hf/W (*39*). Another possible cause for the enrichment in μ^182^W displayed by the ISB samples is fractionation of mantle Hf/W during magma ocean crystal-liquid differentiation processes and the more incompatible behavior of W relative to Hf (*10*). Variants of this model have been invoked to account for positive μ^182^W anomalies in the 2.8-Ga Kostomuksha komatiites (*39*) and the existence of negative μ^182^W anomalies in the 3.55-Ga Schapenburg komatiites (*12*). In the case of the ISB, a silicate liquid-melt differentiation scenario is consistent with the preservation of positive μ^142^Nd anomalies (*10*). The admixture of material from the outer core is another mechanism that has been proposed to explain μ^182^W heterogeneity in modern basalts (*11*); however, this process only appears to account for negative μ^182^W anomalies, rather than the μ^182^W excesses observed in the ISB. A final possibility is that Hf/W fractionation took place as a consequence of FeO disproportionation in the lower mantle to Fe^3+^ and metallic iron, and the extraction of some of this metallic iron (and W) into the core while ^182^Hf was still extant (*5*, *14*). This scenario can potentially be combined with internal silicate differentiation events, e.g., magma ocean crystallization and the formation of refractory cumulates, to account for the presence of μ^142^Nd excesses in the ISB and other ancient mantle melts.

The positive correlation between μ^182^W and δ^57^Fe suggests that Fe isotope partitioning must be associated with the events that caused Hf/W fractionation. Consequently, we may be able to use the Fe isotope systematics of the Isua samples to differentiate between the different mechanisms proposed to explain their μ^182^W enrichments. The first two scenarios, involving mixing of late-accreted chondritic material into the mantle and subsequent high-pressure metal-silicate segregation, are considered unlikely as they do not predict any relationship between μ^182^W and δ^57^Fe, as minimal Fe isotope fractionation is expected to take place between silicate and metallic melts at high pressures (*16*, *20*). The third possibility, of silicate solid-liquid differentiation, is plausible although we note that existing data suggest that the magnitude of Fe isotope fractionation between bridgmanite, ferropericlase, and silicate melt may be limited in nature (*15*, *19*). However, the fourth potential scenario, of disproportionation (*5*), does predict a positive correlation between μ^182^W and δ^57^Fe, due to the incorporation of isotopically light Fe into metallic iron relative to bridgmanite (and potentially, ferropericlase; *15*) and the removal of this metallic iron to the core (*14*). The magnitude of Fe isotopic fractionation associated with this process, as constrained by experimental data, is likely to be significantly larger than in cases where only silicate liquids are involved although further experimental studies are clearly desirable. On this basis, we consider this to be the most likely explanation for the coupled μ^182^W excesses and Fe isotope signatures of the Isua samples. Furthermore, the positive correlation between μ^182^W and δ^57^Fe provides evidence for disproportionation and segregation of metallic iron from the lower mantle within the lifetime of ^182^Hf, i.e., within the first ~45 Ma of Earth history and hence for the presence of an oxidized lower mantle immediately following magma ocean cooling and crystallization in the aftermath of the Moon-forming giant impact.

Our findings also have implications for recent studies, which have revealed that OIB from Hawaii, Samoa, Iceland, and Pitcairn display negative μ^182^W anomalies that correlate negatively with ^3^He/^4^He, suggesting that the negative-μ^182^W domain resides in the primordial, largely undegassed lower mantle (*8*). With the exception of the large igneous provinces sampled by Baffin Island, and, possibly, Ontong Java Plateau (*40*) excesses in μ^182^W are restricted to rocks >2.7 Ga in age [e.g., (*10*–*12*, *38*, *39*)]. While the negative μ^182^W anomalies associated with mantle plumes can be interpreted in terms of contributions from the outer core (*11*) or the incorporation of low Hf/W metallic iron produced by disproportionation of FeO in the lower mantle (*8*), the sampling of positive-μ^182^W mantle by pre–2.7-Ga melting events as well as rare Phanerozoic large igneous provinces remains enigmatic. One possibility is that this reflects the sampling of highly refractory material by ancient, presumably hotter mantle melting events (*41*). On the other hand, our models suggest that the positive-μ^182^W ISB component was formed by eutectic melts of a cumulate residue initially formed during the crystallization of a of magma ocean (Fig. 2E). These second-stage melts would form fusable, pyroxene-rich mineralogies when melted in the upper mantle. If this is the case, then it may be that most of these positive-μ^182^W domains were simply melted and removed from later sampling early in Earth history. The corollary to this is that the bridgmanite-dominated residue produced in equilibrium with these magma ocean cumulate melts would be highly refractory and, if they had accumulated metallic iron (potentially as a consequence of the extraction of the second-stage eutectic melt), would display negative μ^182^W and, theoretically, light δ^57^Fe. Further systematic μ^182^W and δ^57^Fe studies of OIB coupled with those of ancient and modern high mantle potential temperature melts are required to test this hypothesis.

## MATERIALS AND METHODS

### Experimental design

The objective of this study was to determine whether ancient rock samples preserved Fe isotope evidence for a deep-seated source component. The study was composed of two key elements: (i) the Fe isotope analysis of a series of well-characterized ancient mantle melts and (ii) the phase equilibria modeling of melting processes operating in the lower and upper mantle, with the objective of matching the Fe isotope, trace element, and published μ^182^W, ^143^Nd/^144^Nd, and ^176^Hf/^177^Hf data.

#### Iron isotope analysis

Iron isotope analyses were carried out on whole-rock powders used in previous studies (*9*, *10*) that represent ~1.5 kg of powdered and homogenized sample. Approximately 50 mg of rock powder was weighed out, and dissolution, iron purification, and isotopic analyses were undertaken at the University of Cambridge following standard methods [e.g., (*21*, *32*)]. Iron yields were quantitative and chemistry blanks were < 3-ng Fe and negligible compared to the quantities of sample Fe (> 200-μg Fe) processed. Isotopic analyses were performed in medium resolution (*M*/Δ*M* > 8000) on a multiple-collector inductively coupled plasma mass spectrometer (Thermo Neptune Plus) in wet plasma mode using a Scott double-pass quartz spray chamber. Sample solutions consisted of 6–parts per million (ppm) Fe in 0.1 M HNO_3_. Instrumental mass bias was corrected for by sample-standard bracketing where sample and standard Fe beam intensities (typically 35- to 40-V ^56^Fe) were matched to within 5%.

#### Statistical analysis of isotopic data

Errors on Fe isotope compositions (Table 1) are quoted as 2 SD of the mean of multiple analyses; unless otherwise stated, all analyses are for a single sample dissolution analyzed across several different analytical sessions. Mass dependence, reproducibility, and accuracy were evaluated by analysis of an in-house “iron chloride” salt standard (“FeCl_2_”) previously analyzed in other studies [e.g., (*21*) and references therein] in conjunction with international rock standards. The values obtained were FeCl_2_, δ^57^Fe = −1.04 ± 0.04‰; δ^56^Fe = −0.71 ± 0.03‰, 2 SD, *n* = 45; BHVO-2, δ^57^Fe = 0.19 ± 0.06‰; δ^56^Fe = 0.13 ± 0.03‰, 2 SD, *n* = 6; BIR-1, δ^57^Fe = 0.06 ± 0.02‰; δ^56^Fe = 0.04 ± 0.04‰, 2 SD, *n* = 4; BIR-1b (replicate dissolution), δ^57^Fe = 0.06 ± 0.04‰; and δ^56^Fe = 0.05 ± 0.03‰, 2 SD, *n* = 4. All standard values obtained are in excellent agreement with those of previous studies [e.g., (*21*–*22*)]. It should be noted that the FeCl_2_ standard is a pure Fe solution and did not require column processing, whereas the rock standards were processes through column chemistry at the same time as the samples.

### Geological setting and samples

The ISB of southwest Greenland is one of the most highly studied Archean terrains [e.g., (*9*–*10*, *34*, *38*, *42*)]. It predominantly consists of amphibolites derived from basaltic intrusive and extrusive rocks, precipitated sediments such as banded iron formations, felsic intrusive rocks, and ultramafic units (*43*). The samples that we have chosen to study are tholeiitic amphibolites (formerly pillow lavas and metagabbros) from the northern terrane of the ISB studied by Rizo *et al*. (*9*). They were metamorphosed at amphibolite facies conditions and consist primarily of hornblende (tremolite and actinolite). No primary mineral assemblages are preserved, although in some rare low-strain cases, primary magmatic fabrics and features (e.g., deformed pillow margins and relict gabbroic textures) can still be identified. The minimum age of these samples is constrained by U-Pb zircon ages of 3690 to 3720 Ma for tonalites that intrude into the amphibolites (*44*). All of the studied samples have been characterized for major and trace elements as well as radiogenic ^147,146^Sm-^143,142^Nd and ^176^Lu-^176^Hf (*9*) and ^182^Hf-^182^W (*10*). Samples were selected on the basis of closed-system isochron ^147^Sm-^143^Nd and ^176^Lu-^176^Hf behavior, low loss-on-ignition (LOI) values [0.20 to 1.18%; (*9*)] and the absence of significant Ce anomalies, which can be indicative of seawater alteration. We selected all the samples that had decoupled Hf-Nd isotope systematics, i.e., those which defined the 3.72-Ga Lu-Hf and Sm-Nd isochrons of Rizo *et al*. (*9*), and for which W isotope data were also available (*10*). Only five samples fitted these criteria, and all were analyzed. We incorporated a sixth sample, 00-012, only analyzed in the study by Rizo *et al*. (*9*), on the basis of its chemically pristine (LOI = 0.20) composition.

As with any ancient mantle-derived magmas, the tectonic setting of the ISB amphibolites is difficult to constrain. Their major and trace element chemistry has been previously interpreted [e.g., (*9*)] to suggest derivation by partial melting of a broadly peridotitic lithology (olivine + pyroxene) in the upper mantle. While a subduction setting has been proposed for some ISB metabasalts with boninitic-like affinities (*34*), these samples are distinct to the ISB amphibolites studied here, which have tholeiite-like affinities and which are considered to represent oceanic crust formed in some form extensional tectonic setting as they do not have island arc basalt-like trace element signatures (*45*). It has been proposed that the formation of boninitic-like samples in a subduction setting would have involved the metasomatism of their mantle source region by melts or fluids from the subducting slab and, potentially, the preferential addition of Nd relative to Sm, Lu, and Hf (*34*), which could potentially explain some of the ^143^Nd/^144^Nd and ^176^Hf/^177^Hf decoupling observed in these samples (*34*). However, as pointed out by Hoffmann (*34*), even the samples with the most depleted ^143^Nd/^144^Nd signatures (i.e., those that display minimal evidence for metasomatism) are still decoupled with respect to ^176^Hf/^177^Hf. Although these arguments do not apply directly to our samples, they nonetheless make the case for the preservation of deep-seated mantle isotopic heterogeneities irrespective of tectonic setting.

### The influence of posteruptive Fe mobility and magmatic differentiation on Fe isotopes

#### Posteruptive Fe and W mobility

Ancient rock suites inevitably experience substantial metamorphic and metasomatic processing. As discussed above, the 3.72-Ga Isua metabasalts have been metamorphosed to amphibolite grade, and no primary mineral assemblages are preserved. Although we carefully selected samples on the basis of closed-system Lu-Hf and Sm-Nd isochron behavior, low LOI, and the absence of Ce anomalies, it is nonetheless important to demonstrate that these samples did not experience any significant posteruptive Fe mobility that would have impacted their Fe isotope compositions.

**Fig. 4:**
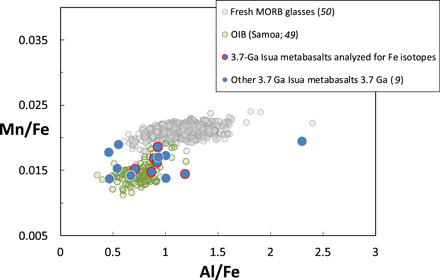
Covariation between bulk rock Al/Fe and Mn/Fe. Ratios were calculated from published major element oxide data for these samples assuming that all iron is present as Fe^2+^.

Postemplacement Fe mobility should manifest as near-vertical trends on a Mn/Al versus Mn/Fe plots (*46*–*48*). These are not observed (Fig. 4), suggesting that the ISB samples have remained a closed system with respect to Fe and that their Fe isotope compositions record magmatic values. In Fig. 4, the ISB samples form a broadly horizontal array, as expected for magmatic differentiation. Fresh MORB glasses and high-Fe Samoa basalts are also plotted in Fig. 4 for comparison [data sources: (*9*, *49*, *50*)]. Both the Samoa samples and the MORB glasses define near-horizontal arrays, as expected for magmatic differentiation, but the Samoa samples plot at consistently lower Mn/Fe than MORB due to the presence of greater proportions of garnet-bearing lithologies (either pyroxenite or lherzolite) in their mantle source region relative to MORB. The ISB samples define a similarly shallow array at intermediate Mn/Fe relative to MORB and Samoa, and this is considered to reflect the relatively minor involvement of garnet in their petrogenesis (i.e., the ambient mantle, μ^182^W = 0, low Lu/Hf component inferred from Fig. 1). Together, these observations provide strong evidence for the relative lack of postemplacement Fe mobility in these rocks. The observation that unusual heavy δ^57^Fe values are restricted to the most primitive [>8 weight % (wt %) MgO] samples that have experienced the least amounts of crustal processing and fractional crystallization serves to reinforce this point. Last, all samples have high Fe contents (Fe_2_O_3_^t^ > 10 wt %) and should therefore be extremely resistant to subsequent perturbation of their Fe isotope compositions.

Posteruptive processes, such as metamorphic fluid infiltration, have also been linked to the decoupling of Nd-Hf systematics and to the distribution of W anomalies in ancient rocks (*33*, *51*). For example, the range in Isua μ^182^W could be regarded as a mixing array between a typical, radiogenic, Archean component [incompletely overprinted by the late veneer; e.g., (*38*)] and a more modern source, potentially created by late metamorphic fluid overprinting, as W is mobile in oxidizing fluids (*33*). However, no correlations exist between δ^57^Fe and W concentration, or W/Th ratio, which is widely used as proxy for the mobility of W relative to other elements of similar incompatibility (*51*). Moreover, fluids released during late-stage magmatic processes (*52*), hydrothermal alteration (*53*), and the prograde metamorphism of ultramafic rocks (*54*) have all been shown to have light δ^57^Fe values. Therefore, this particular scenario cannot account for either the heavy-δ^57^Fe Isua-positive μ^182^W endmember in the ISB suite that we interpret as our “bridgmanite component,” nor does it account for our other endmember, which has a “normal” mantle-like δ^57^Fe and no μ^182^W anomaly.

#### Metamorphic and metasomatic decoupling of Hf-Nd isotopes

While the decoupled Hf-Nd signatures of the ISB have been interpreted here and in (*9*) as a primary source feature, the decoupling of these isotope systems is a well-known phenomenon and has been linked to the greater fluid mobility of Nd relative to Sm, Lu, and Hf (*55*). As mentioned earlier, the preferential mobility of Nd in fluids and the selective addition of Nd to target rocks have also been proposed as a mechanism that can partially explain the decoupled Hf-Nd isotope systematics of Isua metabasalts and ultramafic rocks (*34*). However, as noted in (*34*), the most depleted samples, where there is no evidence for metasomatic enrichment, still display Hf-Nd isotope decoupling, suggesting that this may be a primary mantle source feature. Other cases of Hf-Nd coupling also exist. For example, Hammerli *et al*. (*56*) related Hf-Nd resetting in Eoarchean tonalites to the metamorphic destruction and recrystallization of allanite, resulting in the loss of unradiogenic Nd from that phase, generating a whole rock with a higher present-day ^143^Nd/^144^Nd ratio relative to that predicted from its ^176^Hf/^177^Hf. It has also been shown that seawater-peridotite interaction has the potential to decouple Hf-Nd isotopes in ultramafic rocks (*57*) by modifying Nd isotopes with minimal change in Hf isotopes. Similarly, mantle metasomatism has also been shown to reset Nd-Hf isotopes through the preferential mobility of Nd, where the deepest, most fertile lithosphere is most strongly affected while shallow, depleted oceanic lithosphere (with radiogenic Hf) remains relatively unmodified (*55*). However, there is little evidence that any metamorphic and metasomatic processes that (may) have contributed to the Hf-Nd isotope decoupling observed in the ISB also contributed to their Fe isotope signatures. First, the Fe concentrations of fluids are extremely low because of the minimal solubility of Fe in fluid phases (*58*), and second, as discussed above, all Fe isotope studies that have explored fluid-related modification of igneous rock Fe isotope signatures have demonstrated that fluid phase are characterized by light δ^57^Fe, ruling out these processes as an explanation for the unusual heavy δ^57^Fe signatures observed here. Last, the mantle xenoliths displaying Hf-Nd decoupling studied in (*55*) have also been analyzed for their Fe isotope compositions (*21*). These samples display strong correlations between δ^57^Fe and Hf isotope composition that are indicative of melt extraction processes. Critically, no correlations between δ^57^Fe and indices of metasomatic enrichment were present in these samples (*21*), demonstrating the relative immobility of Fe during these processes.

#### Fe isotope fractionation during magmatic differentiation

Stable Fe isotope fractionation during magmatic differentiation is a well-documented phenomenon [e.g., (*13*, *17*, *29*–*30*, *33*, *34*, *41*, *47*–*49*, *59*–*61*)] that is driven by differences in bond strength between silicate minerals and melt. Theoretical studies and empirical observations predict that isotopically light iron will be associated with Fe^2+^-bearing phases, such as olivine, while Fe^3+^-bearing phases, such as magnetite and clinopyroxene, should be isotopically heavy. Silicate melts are also predicted to be isotopically heavy relative to all upper mantle solid silicate and oxide phases, because of the incompatibility of Fe^3+^ relative to Fe^2+^ and the preferential concentration of Fe^3+^ into partial melts relative to cumulate residues. This prediction has been confirmed in several studies of cogenetic magmatic suites from different tectonic settings, where δ^57^Fe is observed to increase with indices of magmatic differentiation until magnetite saturation at ~4 wt % MgO (*58*–*61*). In these suites, the most primitive samples display the lightest δ^57^Fe values and δ^57^Fe then increases with magmatic differentiation and the removal of isotopically light (relative to the melt) olivine, spinel, and pyroxene phases. In some studies [e.g., (*31*, *62*)], the effects of magmatic fractionation on sample δ^57^Fe have been “corrected” for by incrementally adding olivine, with a set Fe isotope fractionation factor between olivine and melt, back to the melt until a bulk-rock Mg# of 0.74 is achieved.

The impact of magmatic fractionation on the Fe isotope compositions of the ISB can be evaluated using the plot of δ^57^Fe versus MgO (Fig. 1A) and comparing the trend defined by the ISB samples to that of MORB (*29*). It can be seen that the ISB define a trend opposite to that of MORB, where the most primitive ISB (ca. 8.9 wt % MgO) has the heaviest δ^57^Fe values and the most evolved samples the lightest δ^57^Fe values. The role of magmatic fractionation in generating the δ^57^Fe values of the ISB samples was also evaluated by correcting the measured δ^57^Fe values back to a primitive composition (Mg# = 74, to be in equilibrium with Fo_90_ olivine) using the approach of (*62*) discussed above. As seen in Table 1, this correction has the greatest impact on the most evolved samples and serves to decrease their δ^57^Fe values whereas the impact on the least evolved samples is minimal. A plot of raw (uncorrected) sample δ^57^Fe versus Mg# is shown in Fig. 3A, and plots of “primary” δ^57^Fe values (corrected by olivine addition; see Results for discussion) against μ^182^W, Lu/Hf, and Zr/Nd in Fig. 3 (B to D). Plots of both uncorrected and corrected δ^57^Fe values against μ^142^Nd are shown in Fig. 3 (E and F). These plots serve to demonstrate that the impact of magmatic differentiation on the δ^57^Fe values of the ISB samples and their correlations with other isotopic and trace element signatures is minimal. It is worth noting that while Mg# = 74 is a reasonable assumption of the composition of a primary melt in equilibrium with a peridotitic mantle lithology, it may not be appropriate for more pyroxene-rich lithologies, such as that hypothesized to form from lower mantle material that is transported to the upper mantle. These corrections to Mg# = 74 should therefore be seen as purely illustrative.

### Phase equilibria models and calculating the composition of lower mantle melts and cumulates

Our model to account for the observations described in the main text consists of three stages:

1) The generation of a cumulate composed largely of bridgmanite, consistent with the Lu-Hf and Sm-Nd decoupling observed in the ISB (*9*);

2) Eutectic-like melting of this cumulate, subsequent melt extraction, and solidification;

3) Transport of this solidified cumulate melt to the upper mantle where it melts and contributes to the Isua metabasalts.

In the main text, we demonstrate that this multistage process can explain the observed isotope and trace element systematics of the ISB. Here, we explain these calculations in more detail and demonstrate the likely melting behavior of these lower mantle–derived lithologies once transported to the upper mantle. We use thermodynamic models to calculate the upper mantle phase-equilibria of the lithologies, in the NCFMASCrO system.

We first describe how our lower mantle model works, then explain how we applied it to making quantitative predictions for the first two stages of our model. Last, we describe our upper mantle melting and thermodynamic calculations.

#### A model for lower mantle phase chemistry and the formation of a bridgmanite cumulate

We assume that the magma ocean has the major element chemistry of model CI chondrite after core formation (*63*). The trace element concentrations of the BSE were taken from (*27*) to maintain consistency with previous modeling of these samples (*9*). For computational convenience, we assume that the mass proportion of liquid is much greater than the mass proportion of cumulate crystals (0.1%). The cumulate is assumed to be composed of 95% bridgmanite and 5% calcium perovskite as required by the Sm-Nd and Lu-Hf constraints (*9*). For simplicity, we do not include ferropericlase. We note that the addition of ferropericlase had no significant effect on the trace element and isotope predictions because we assume that the principal driver of Fe isotope fractionation is between bridgmanite and Fe metal (*14*), rather than between bridgmanite and ferropericlase, which in any case is predicted to be minor (*15*). While the addition of ferropericlase would make a difference to the upper mantle phase equilibria, this is out of the scope of the present study.

To estimate the major element composition of the lower mantle cumulate and cumulate-derived melt in the NCFMASCrO system, the composition of each crystalline phase and the magma with which they equilibrate must be estimated. A thermodynamic model that links crystalline mantle phases and magma at lower mantle conditions does not yet exist for the NCFMASCrO system, and so we take an empirical approach. We note that while Stixrude and Lithgow-Bertelloni (*64*) have produced a comprehensive thermodynamic model for mineral equilibria in the lower mantle, their model lacks a magma phase. Boukaré *et al*. (*65*) also calibrate a mineral-magma model for the lower mantle, but in the MgO-FeO-SiO_2_ system, and so their model cannot be used for modeling equilibria involving Ca perovskite.

We start with pure bridgmanite (MgSiO_3_), calcium perovskite (CaSiO_3_), and ferropericlase (MgO) compositions. For each element in NCFMASCrO that does not appear in the phase’s formula, a partition coefficient is used to calculate the extent of solid solution. Depending on which crystal site the element is most likely to reside in Table 3, atoms are subtracted from the pure formula so that stoichiometry and mass balance is maintained. For example, the moles of Mg in ferropericlase is given by

$$X_{\text{Mg}}=1-(X_{\text{Si}}+X_{\text{Ca}}+X_{{\text{Fe}}^{2+}}+X_{\text{Al}}+X_{\text{Cr}}+X_{\text{Na}})$$

where *X*_Si_, *X*_Ca_, *X*_Fe^2+^_, *X*_Al_, *X*_Cr_, and *X*_Na_ are calculated from the molar partition coefficients given in Table 3. The partition coefficients were calculated from experimental phase compositions (*25*–*27*). The predicted major element chemistry of the cumulate is shown in Table 4.

**Table 3:** The molar partition coefficients used to calculate the quantities of elements on each crystallographic site in bridgmanite (Bg), ferropericlase (Fp), and calcium perovskite (Ca-Pv). See Materials and Methods text for more details. Partition coefficient values are from (1) Walter *et. al.* (2004) [*27*] Exp. 62; (2) Liebske *et. al.* (2005) [*26*] Exp. H2033; (3) Corgne *et. al.* (2005) [*25*] Exp. H2020b.

**Element**	**Bg (A)**	**Bg (B)**	**Fp (A)**	**Ca-Pv (A)**	**Ca-Pv (B)**
Si	0	Stoichiometric	0.005^2^	0	Stoichiometric
Mg	Stoichiometric	0	Stoichiometric	0.11^3^	0
Ca	0.11^1^	0	0.012^2^	Stoichiometric	0
Fe^2+^ and Fe^3+^*	0.67^1^	0	1.25^2^	0.1^3^	0
Fe^3+^	See note*	See note*	0	See note*	See note*
Al	0.65^1^	0.65^1^	0.36^2^	0.55^3^	0.55^3^
Na	0.12^1^	0	0.5^2^	0.37^3^	0
Cr	0.26^1^	0.26^1^	1.24^2^	0.21^3^	0.21^3^

*It is assumed that Fe^3+^ and Fe^2+^ are incorporated in the ratio 6:4, with Fe^3+^ distributed evenly between the two perovskite sites.

**Table 4:** The major element chemistry of the lithologies calculated here, with KLB-1 for comparison. All quantities are in mole percent. LM, lower mantle.

**Lithology**	**SiO_2_**	**Al_2_O_3_**	**CaO**	**MgO**	**FeO**	**Fe_2_O_3_**	**Na_2_O**	**Cr_2_O_3_**
KLB-1 (*69*)	38.49	1.78	2.82	50.57	5.69	0.10	0.25	0.11
Cumulate (no Fe^0^ retained)	48.31	2.45	2.48	44.55	1.20	0.90	0.04	0.08
Cumulate (most Fe^0^ retained)	47.88	2.42	2.46	44.15	2.97	0.01	0.04	0.08
LM melt (no Fe^0^ retained)	48.49	2.33	14.78	32.68	0.91	0.68	0.06	0.07
LM melt (most Fe^0^ retained)	48.16	2.32	14.68	32.46	2.26	0.01	0.06	0.07

We assume an Fe^3+^/ΣFe of 0.6 in bridgmanite, in accordance with experiments (*66*). We assume that any Fe incorporated into calcium perovskite also has an Fe^3+^/ΣFe of 0.6, which is reasonable as Fe^3+^ is likely incorporated into the perovskite structure by a coupled substitution replacing Si^4+^ with Al^3+^, while Mg^2+^ is exchanged with Fe^3+^ (*67*). While this mechanism places the majority of Fe^3+^ on a single site, the coupled substitution means the fraction Si^4+^ should decrease with increasing Fe^3+^ and Al^3+^ fraction. To achieve this behavior without adding complexity to our model, we allow Al^3+^ and Fe^3+^ to partition onto both sites in equal proportions.

As, for simplicity, our initial magma composition contains no initial Fe^3+^ (hence provides a minimum estimate of melt Fe^3+^), upon bridgmanite crystallization, iron metal is generated according to the reaction (*5*)

$${\text{Fe}}^{2+}\to \frac{2}{3}{\text{Fe}}^{3+}+\frac{1}{3}{\text{Fe}}^{0}$$

From this model, we obtain the major and trace element compositions of the residual cumulate and associated melt phase. The bulk composition that we obtain for the residual cumulate then defines the bulk composition of the cumulate that is equilibrated with melt in the following section. A demonstration of the model is provided as an Excel file in the Supplementary Materials.

While metal-silicate segregation and generation and partial removal of Fe metal from this cumulate are an important part of our model for determining the proportion of Fe metal present in our lower mantle component, it is important to stress that our model does not directly incorporate Fe isotope partitioning fractionation factors, because of the fact that these are so poorly constrained at lower mantle conditions. Instead, we assume that our cumulate component has a heavy δ^57^Fe signature that we simply fix at 0.30‰. This value is demonstrably lower than that predicted for bridgmanite–Fe metal partitioning [estimated to be at least ~0.6‰ for ^57^Fe/^54^Fe at 24 GPa and 1850 K (*14*)]. We have used this low, conservative estimate to take into account the complex combined effects of temperature and incomplete Fe metal segregation on the Fe isotope composition of the cumulate.

#### Eutectic melting of the bridgmanite cumulate

We then envisage a eutectic-like melting reaction in the lower mantle. The trace element chemistry of the derivative melt is calculated by equilibrating a melt with a 100% bridgmanite residue, using the bulk composition calculated in the previous step and assuming batch melting. For the major elements, we assume that the melting reaction consumes all the calcium perovskite (5 wt % of the cumulate), along with a further 10 wt % bridgmanite, achieving a total melt fraction of 15 wt %. For simplicity, we assume that the residual bridgmanite composition is identical to that calculated in the previous step. These proportions were chosen to provide a good fit to the trace element ratio mixing hyperbolae (Fig. 1) following the third stage of our model. The predicted major element chemistry of the melt and the resulting solid lithology are given in Table 4.

#### Upper mantle melting and magma mixing

Following solidification and transport to the upper mantle, we then calculate the trace element concentrations of magmas composed of a mixture of melt from the cumulate-derived lithology and melt from a depleted upper mantle component (*68*). Again, for simplicity, we assume batch melting and use the partition coefficients calculated in (*68*) for typical upper mantle lherzolite melting. As discussed below, we assume comparatively high-fraction melts of mixture of melt from the cumulate-derived lithology and melt from a depleted upper mantle component. While the mineralogy of the cumulate-derived component will be more akin to a pyroxenite than a lherzolite, little difference will be observed for high-fraction batch melts. Although the degree of melting involved in producing the ISB suite is unknown, the assumption of high melt fractions is reasonable given the high potential temperatures of Archean mantle and the fusibility of the cumulate-derived pyroxenite lithology. We also note that the high degree of melting required in our batch calculations could be obviated by polybaric fractional melting with incomplete aggregation of the deepest melts. Therefore, the degree of melting is effectively a free variable trading off with assumptions about the style of melting.

Applying these models, we find a good fit to the mixing hyperbolae in Fig. 1 (B to D) when we mix a 50% melt of the more fusible pyroxenite-like, cumulate-derived, lithology with a 30% melt of depleted upper mantle [note that while the Isua samples are certainly not primordial melts, magmatic differentiation will not significantly affect their μ^182^W and δ^57^Fe or trace element ratio systematics and hence the mixing arrays seen in Fig. 1 (B to D)]. The values of μ^182^W and δ^57^Fe taken for the cumulate-derived mixing endmember were 20 ppm and 0.30‰, respectively. We assume no Fe isotope fractionation during this melting event, given the high temperatures and large melt fractions. The choice of μ^182^W and δ^57^Fe endmember values was conservative and, as discussed in the main text, informed by the qualitative expectations of μ^182^W variability in early magma ocean cumulates [from both Hf/W partitioning between metal and silicate and W isotope data for large-volume mantle melts such as komatiites; e.g., (*11*, *12*)] and the magnitude of Fe isotope fractionation between bridgmanite and iron metal [~>0.6‰; (*14*)].

#### Calculating the upper mantle phase equilibria and isotope fractionations

To explore the melting behavior of our hypothesized mantle lithologies, and whether the upper mantle melting process can explain our observation of heavy δ^57^Fe, we construct pseudosections and calculate mineral chemistry along a mixing line between KLB-1 lherzolite and both the cumulate derived from the first stage of the model and its eutectic-like melt (Table M2). The calculations are performed in the NCFMASCrO system using thermocalc v3.40 (*35*), the dataset ds-622 (*36*), and the a-X relations of (*37*). We use the KLB-1 composition of (*69*). All calculations were performed at 50 kbar.

Following calculation of the phase field boundaries, thermocalc was run over a grid to identify phase proportions and chemistry. Using the equations presented by Sossi and O’Neill (*13*), we estimate the force constants for Fe─O bonds for each mineral site hosting Fe in olivine, clinopyroxene, orthopyroxene, and garnet. The parameters used in our calculations are listed in Table 5. We use the force constant estimated for basaltic melts (194 N m^−1^) in (*18*) to estimate the fractionation factors between each mineral site and melt and then estimate the deviation of δ^57^Fe in the magma with respect to the bulk composition (δ^57^Fe = 0, for simplicity). The calculations demonstrate that upper mantle melting processes impart minimal change in δ^57^Fe, further emphasizing that the driver for δ^57^Fe variation must be the initial δ^57^Fe of the source region, which, we assume, is inherited from the initial cumulate stage, where it was produced by disproportionation and the removal of an Fe isotopically light metal phase.

**Table 5:** Parameters used in calculating of the force constants for each mineral site. All other constants are as used in (*13*). The ionicity is set to 0.36. For each equilibrium calculation, *K*_T_ is calculated for each mineral using the molar proportions of Fe^2+^ and Fe^3+^ on each site, as determined using THERMOCALC.

**Mineral**	**Species**	**Site**	**Cation** **coord.**	**Bond length**	**Oxygen** **coord.**	**No. in formula**	***K*_f_** **(N m^−1^)**	***K*_T_** **(N m^−1^)**
Spinel	Fe^2+^	M	6	2.15	4	2	43	178
Spinel	Fe^2+^	T	4	2.00	4	1	79	221
Spinel	Fe^3+^	M	6	2.025	4	2	76	319
Spinel	Fe^3+^	T	4	1.875	4	1	145	401
Garnet	Fe^2+^	M1	8	2.291	4	3	26	147
Garnet	Fe^3+^	M2	6	2.024	4	2	77	319
Olivine	Fe^2+^	M	6	2.1685	4	1	42	173
Orthopyroxene	Fe^2+^	M1	6	2.135	3.66	1	48	198
Orthopyroxene	Fe^3+^	M1	6	2.118	3.66	1	73	304
Orthopyroxene	Fe^2+^	M2	6	2.228	3.33	1	46	192
Clinopyroxene	Fe^2+^	M1	6	2.14	3.66	1	47	197
Clinopyroxene	Fe^3+^	M1	6	2.033	3.66	1	83	344
Clinopyroxene	Fe^2+^	M2	6	2.526	3.75	1	28	117

**Fig. 5:**
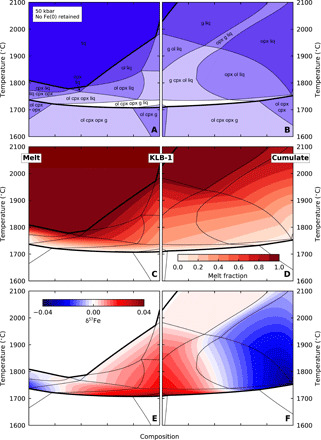
Model results from upper mantle phase equilibria calculations, with efficient Fe metal removal in the lower mantle. Results are shown from lithologies intermediate between KLB-1 peridotite (middle), the lower mantle bridgmanite cumulate (right), and the lower mantle melt derived from the cumulate (left). Efficient Fe metal removal in the lower mantle results in an Fe^3+^-rich upper mantle lithology. (**A** and **B**) Phase assemblages as a function of temperature and composition; the shading indicates field variance. The solidus and liquidus are indicated by thick lines. (**C** and **D**) Fraction of melt present. (**E** and **F**) Deviation of δ^57^Fe of the liquid phase from the bulk δ^57^Fe. The persistence of g + liq to very high T (with very high melt fraction) is an artifact of the THERMOCALC model.

**Fig. 6:**
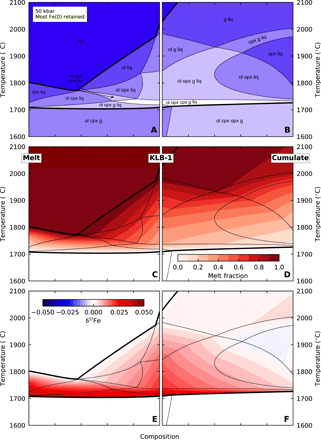
Model results from upper mantle phase equilibria calculations with inefficient Fe metal removal in the lower mantle. In this case, sufficient Fe metal is retained so that no Fe^3+^ is present in the upper mantle cumulate and cumulate-derived melt assemblages. (**A** and **B**) Phase assemblages as a function of temperature and composition; the shading indicates field variance. The solidus and liquidus are indicated by thick lines. (C and D) Fraction of melt present. (E and F) Deviation of δ^57^Fe of the liquid phase from the bulk δ^57^Fe. The Fe ^3+^ content of KLB-1 is unchanged. The persistence of g + liq to very high T (with very high melt fraction) is an artifact of the THERMOCALC mode.

A large uncertainty in performing these calculations is the extent of iron metal extraction from the cumulate while in the lower mantle. We consider two endmembers (Table 4): complete metal loss (Fig. 5) and sufficient iron retention for all Fe^3+^ to be reduced to Fe^2+^in the upper mantle (Fig. 6).

## SUPPLEMENTARY MATERIALS

Supplementary material for this article is available at http://advances.sciencemag.org/cgi/content/full/7/11/eabc7394/DC1
